# The Physician in Literature

**Published:** 1907-03

**Authors:** 


### THE PHYSICIAN IN LITERATURE.

This phrase has become current with the increase of contributions of medical origin, and has generally been applied to ventures of physicians into general literature. The surpassing genius with the pen of Oliver Goldsmith and Oliver Wendell Holmes has given to them a literary fame in the shadow of which their medical lives have been eclipsed. But it is difficult for physicians to relinquish the claim upon these delightful writers as colleagues. Among medical authors of the present day Weir Mitchell is facile princeps, and of all who have taken this dual place of novelist and physician, he alone has maintained supremacy in either branch of intellectual endeavor, and is known to the world no less as a physician of highest scientific attainments than as an author of rarely erudite works.

The phrase "The Physician in Literature" has another application when used in description of the doctor as seen by his patients, and many medical characters in fiction have become famous. Ian MacLaren's William MacLure appeals most forcibly to our sympathies, and we like to think of him as the type. To clear the ambiguity of the phrase and to meet all its requirements, implied and actual, however, have been left for an Albanian. In the Christmas Argus of this season, under the title "A Hero of the Hills: Chronicle of a Mountain Medical Parish, By Frederic C. Curtis, M. D.," is an excellent piece of literary work of a physician, by a physician and for—everybody. True to life, and free from even the accentuation which is the privilege of the novelist, this biographical sketch brings into delightful harmony the relations of the hard service of a country practitioner with the romance which is too often regarded as solely a figment of the imagination. Before the vision of the reader, acquainted with the personalities of this sketch, arises a vivid picture of the conditions of its inspiration. The city consultant, in his luxurious office in the shadow of the majestic capitol of an empire State, to whom the good things of life come easily, looks beyond the artificial parks and magnificent buildings of his environment and recalls the comradeship of a day in the mountain wilderness. After a life-long acquaintance with the masters of his science, medical authors and investigators, physicians prominent in the local and political world, he selects, for the expression of his ideals, the colleague who battles with disease and death, unaided by specialists, in the remote forest. His hero "certainly did realize a love for his work and a devotion to the calling and associations of his profession. All phases of it in the subjects of his care excited his interest. He seemed to seize with avidity the rare chance of talking about them to one who might be appreciative. It was not simply the abstract disease that was pictured, but the environment of each diseased individual, a little child, a poor woman, a workingman, and what they all had to contend with besides the mere disease. He was well informed, and his chronicler was a learner from him, but there was an appeal to him in the humanity of the subject. It was largely poor and unlettered people that he had this work with. Sometimes they were evil, and he could be exceeding severe and rough with them, so that a sort of joy of mastery in his big nature was revealed." Then our author tells how his hero became a doctor; how, without money and with tuberculosis, he sought employment as a guide, was seen and appreciated by medical men and launched upon his benificent career. His life was eventful. "The choppers in the wood camps often injure themselves frightfully with their axes or by falls. One day, getting word of such, he drove several miles to the lake, fastened his horses and rowed across the lake and tramped a mile to the camp. Temporarily fixing up his patient, he had a litter made and the men carried their comrade back to the lake; there he lifted him with his strong, trained arms into the boat, rowed across, picked him up and put him into his carriage, and so brought him home and eventually cured him up. He has no rubber-tired ambulance and hospital with trained nurses at command." "Sometimes, when there was special prevalence of sickness, he would go for one or two weeks without a night in bed. He would get home, have his horses fed and rested, lying, himself, by the kitchen fire for a spell, and then start out in another direction. While his horses plodded along the narrow way he slept. Everybody knew him, for he had followed his work for years, and all helped him on; meeting him sometimes with vehicles going in the opposite direction, they would lead his horses aside, and, having passed, set them on their way again without arousing him. The horses would sometimes stop at a watering trough, and the first passer-by would uncheck them to drink and then start them on while he still slept. All knew the nature of his errands and understood the demands of his physical resources."

Thus does a ray of the Divine dart into the reckless material life of our day. It is with no small satisfaction that the Annals records this excursion into general literature of its first editor—the Nestor of

Albany specialists. More power to his pen. All glory to the Physician in Literature. All honor and love to the Country Doctor!—Editorial, Albany Medical Annals, February, 1907.

